# Supplementary material for: Behavioral correlates of semi-zygodactyly in Ospreys (Pandion haliaetus) based on analysis of internet images
Source: PeerJ. 2019 Feb 5;7:e6243. doi: 10.7717/peerj.6243 (PMC6368007; doi:10.7717/peerj.6243)
Supplement: Supplemental Information 1 — Toe configuration (toe code; 1 = 3 × 1, 2 = 2.5 × 1.5, 3 = 2 × 2) was modeled as a function of grasping scenario (graspscen; free-footed, grasping, perched), object size (objsize; no object [0]–extra-large [4]), the interaction between these latter two variables, and foot identify (footID; left or right, treated as a between-subjects effect), for the complete data set (n = 1882 feet [of 1123 Osprey images]). Main effects were qualitatively unchanged upon successive removals (in order of decreasing P-value) of non-significant interaction terms. Parameter estimates were not supplied here, because none were interpreted from this model. [file peerj-07-6243-s001.docx]

| **Source** | **Type III Wald** χ^2^ | **df** | ***P*** |
| --- | --- | --- | --- |
| graspscen | 150.61 | 1 | <.0001 |
| objsize | .416 | 3 | .937 |
| footID | .346 | 1 | .556 |
| graspscen × objsize | 4.34 | 2 | .114 |
| graspscen × foot | .428 | 1 | .513 |
| foot × objsize | 3.13 | 3 | .372 |
